# AI-Enhanced Predictive Analytics to Optimize Tele-Oncology Implementation in Rural Settings: Scoping Review

**DOI:** 10.2196/78005

**Published:** 2026-07-16

**Authors:** Laiba Husain, Megan Mullins, Bella Etingen, Raaed Mohammed Zafar, Mediha Siddiqui, George L Jackson

**Affiliations:** 1 O’Donnell School of Public Health, UT Southwestern Medical Center, 5323 Harry Hines Blvd, Dallas, TX, 75390, United States, 1 7344863806; 2 Research and Development Service, Dallas Veterans Affairs Medical Center, Dallas, TX, United States; 3 School of Behavioral and Brain Sciences, UT Dallas, Dallas, TX, United States; 4 Dedman College of Humanities and Sciences, Southern Methodist University, Dallas, TX, United States; 5 Center of Innovation to Accelerate Discovery and Practice Transformation (ADAPT), Durham VA Health Care System, Durham, NS, United States

**Keywords:** artificial intelligence, predictive analytics, tele-oncology, telemedicine, rural health, implementation science, health equity, scoping review

## Abstract

**Background:**

Tele-oncology addresses geographic barriers to cancer care, but implementation challenges persist in rural settings. AI-enhanced predictive analytics offer opportunities for optimizing deployment through personalized, data-driven strategies; however, evidence in rural tele-oncology contexts remains limited, and critical equity considerations remain underexamined.

**Objective:**

This scoping review aimed to map evidence on AI-enhanced predictive analytics in tele-oncology implementation, with particular attention to rural and underserved populations, to identify research gaps and inform implementation science priorities.

**Methods:**

We searched 5 databases (PubMed, Embase, CINAHL, Web of Science, and IEEE Xplore) using 4 concept domains (tele-oncology, rural implementation barriers, AI or predictive analytics, implementation science) from January 2015 through November 2025. Two independent reviewers screened 330 unique records (title or abstract; Cohen κ=0.78), with the principal investigator resolving conflicts. Of 138 full-text reviews (κ=0.82), 4 studies met inclusion criteria. Data extraction captured study characteristics, AI applications, implementation factors, and outcomes. We used narrative thematic analysis to map findings into three themes: (1) the current tele-oncology implementation landscape in rural and underserved settings, (2) potential AI applications addressing implementation challenges, and (3) implementation considerations for AI systems themselves.

**Results:**

Four included studies (1 pilot feasibility study, 1 proof-of-concept validation study, 1 cross-sectional predictive study, and 1 platform development study; published 2019‐2025) demonstrated limited evidence at the intersection of AI, tele-oncology, and rural health equity. Patient characteristics predicted telehealth modality preferences with 86.2% accuracy, revealing that male patients exhibited 66% increased odds of video selection versus female patients (*P*=.004), and urban residents showed 101% increased odds compared to rural counterparts (*P*<.001). Liu et al demonstrated that disadvantaged populations engaged with AI-generated health literacy content 2.52-fold more frequently than nondisadvantaged counterparts. However, all 4 studies documented substantial implementation barriers (patient, provider, organizational, and system levels) persisting despite technological sophistication. Organizational threshold effects, where remote monitoring interventions succeeded with adequate provider capacity but failed under resource constraints—suggest that algorithmic innovations cannot overcome structural limitations in rural facilities. No studies explicitly examined algorithmic bias, cross-population validation, or potential harms in rural contexts. Geographic concentration in high-resource countries (United States n=2, Greece n=1, and Singapore n=1) and limited oncology-specific focus underscore structural gaps in knowledge generation for underserved populations.

**Conclusions:**

Current evidence remains insufficient to support definitive practice recommendations. The observed evidence gap may reflect broader structural inequities in knowledge generation: populations with the greatest implementation challenges appear to remain substantially underrepresented in AI and digital health literature. Future research should prioritize comparative effectiveness studies in authentic rural contexts with implementation science outcomes, equity-centered cross-population validation, specification of translation mechanisms linking AI predictions to implementation strategies, health economic analyses, and mechanistic research on sociotechnical integration factors, ensuring technological innovation reduces rather than perpetuates disparities in cancer care.

## Introduction

### Terminology and Key Definitions

To ensure clarity and consistency throughout this review, we define key terms as follows: we use telehealth as the broader umbrella term encompassing all remote health care delivery using telecommunications technology, including both clinical services (telemedicine) and nonclinical services (tele-education, administrative videoconferencing) [1]. Telemedicine refers specifically to remote clinical services involving diagnosis, treatment, and patient monitoring [1,2]. Tele-oncology denotes the application of telehealth or telemedicine specifically to cancer care delivery, encompassing video consultations for diagnosis and treatment planning, remote symptom monitoring, virtual tumor boards, telephonic follow-up care, and hub-and-spoke models connecting rural facilities with specialized cancer centers [3,4]. AI-enhanced predictive analytics encompasses computational approaches, including supervised machine learning (random forests and gradient boosting), natural language processing, ensemble methods, and deep learning techniques that analyze data to forecast future outcomes, identify patterns, or generate predictions to inform clinical or operational decision-making [5]. Rural is defined broadly to include populations in geographically isolated areas with limited health care access, consistent with heterogeneous definitions across included studies, recognizing that rural classifications vary by country and health care system [6]. Implementation refers to the process of translating evidence-based interventions into routine practice, encompassing activities related to adoption, integration, sustainability, and scale-up of tele-oncology services within health care delivery systems [7].

Tele-oncology has emerged as a critical strategy for expanding access to cancer care, particularly for individuals facing geographic, socioeconomic, or infrastructure-related barriers to traditional in-person services [8,9]. Following rapid implementation during the COVID-19 pandemic [10], tele-oncology has demonstrated feasibility across varied settings and patient populations [4,8,10]. However, substantial implementation challenges persist, including gaps in technological literacy among patients, limitations in broadband infrastructure in rural areas, complexities in workflow integration, barriers to device access, and constraints on health care system resources [11,12].

These implementation barriers disproportionately affect rural and underserved populations, potentially exacerbating existing cancer care disparities [13,14]. Patients with cancer living in rural areas experience lower survival rates and later-stage diagnoses compared to their urban counterparts [15], driven partly by limited access to oncology services, multidisciplinary care teams, and clinical trials [16]. While tele-oncology theoretically addresses geographic access barriers, populations with the greatest potential to benefit from telehealth often demonstrate the lowest usage rates [17]. This implementation gap reflects complex, multilevel barriers spanning individual patient factors (technological literacy, device access, and digital confidence), health care system characteristics (infrastructure capacity, workflow integration, and provider comfort with telehealth), and contextual elements (broadband availability, reimbursement policies, and organizational support) [1,14,18].

Recent advancements in artificial intelligence and predictive analytics present opportunities for addressing these implementation challenges through more personalized, data-driven approaches to tele-oncology deployment [19]. Emerging evidence from adjacent health care domains demonstrates that AI-enhanced systems can accurately predict the preferences of patients for different telehealth modalities based on demographic and clinical characteristics [20], identify individuals at high risk for implementation barriers before they manifest clinically [21], optimize resource allocation by forecasting demand and prioritizing high-need populations [22], and enable remote symptom monitoring with predictive capabilities for early complication detection [23]. These applications suggest potential translational pathways for enhancing tele-oncology implementation, though direct evidence specifically addressing AI applications in rural tele-oncology contexts remains limited.

The integration of AI-enhanced predictive analytics with implementation science frameworks offers a promising approach for optimizing tele-oncology delivery [19,24]. Implementation science provides systematic methods for translating evidence-based interventions into routine practice, with established frameworks for assessing barriers, designing implementation strategies, and evaluating outcomes [7,25]. Recent theoretical work has articulated how AI applications could specifically address implementation science challenges related to speed, sustainability, equity, generalizability, contextual assessment, and causal inference [18,19]. However, the successful translation of AI technologies into clinical practice requires careful attention to implementation considerations, including stakeholder engagement, workflow integration, technical infrastructure requirements, validation across diverse populations, and proactive monitoring for unintended consequences [24].

This scoping review systematically examines current applications of AI-enhanced predictive analytics and digital health technologies across oncology care delivery, with particular attention to implementation considerations relevant for rural and underserved populations. Given the nascent and heterogeneous nature of research at this intersection, we used a scoping review methodology to map existing evidence, identify research gaps, and synthesize findings to inform a suggested research agenda [26,27]. Our specific objectives were to: (1) characterize current applications of AI and predictive analytics in telehealth and digital oncology care; (2) examine empirical evidence on implementation factors, barriers, and facilitators; and (3) identify critical research gaps and future directions for optimizing tele-oncology implementation in rural contexts.

## Methods

This scoping review followed established methodological frameworks [26] and adhered to the PRISMA-ScR (Preferred Reporting Items for Systematic Reviews and Meta-Analyses Extension for Scoping Reviews) guidelines (Checklist 1) [28] and PRISMA-S (Preferred Reporting Items for Systematic Reviews and Meta-Analyses Literature Search Extension) guidelines (Checklist 2) [29] reporting guidelines. Complete adherence documentation is provided in (PRISMA-ScR checklist) and Multimedia Appendix 1 (full search strategy). No published protocol was developed, consistent with exploratory scoping review methodology. The review was not prospectively registered.

Studies were included if they addressed: (1) tele-oncology implementation across any setting, (2) rural health care barriers or implementation strategies relevant to underserved populations, and (3) AI or machine learning applications in health care delivery with transferable insights for oncology. Consistent with Joanna Briggs Institute methodological standards for scoping reviews, which are designed to synthesize primary empirical evidence, systematic reviews, and scoping reviews were excluded from the eligible study designs [26,27]. Where relevant secondary reviews were identified during screening, specifically Aziz et al [30] and Anderson et al [31], these were retained and referenced in the “Discussion” section to contextualize primary study findings within the broader evidence landscape, consistent with accepted practice in scoping review reporting.

Publications were restricted to English-language, peer-reviewed literature from January 2015 through November 2025, capturing recent AI developments and postpandemic tele-oncology expansion. Complete inclusion or exclusion criteria, along with operational definitions, examples, and rationale, are detailed in Multimedia Appendix 2.

We searched 5 databases selected for disciplinary breadth: PubMed or MEDLINE (biomedical literature), Embase (pharmaceutical and European coverage), CINAHL (nursing and allied health), Web of Science (multidisciplinary sciences and implementation research), and IEEE Xplore (computer science and AI technical literature). The search strategy, developed in consultation with a health sciences librarian, integrated 4 concept domains using controlled vocabulary (MeSH, Emtree, and CINAHL headings) and free-text keywords: (1) tele-oncology or telehealth in cancer care, (2) rural health care and implementation barriers, (3) AI and predictive analytics, and (4) implementation science. These domains were combined using Boolean logic to optimize sensitivity while maintaining specificity.

An initial search was completed on April 9, 2025. Following reviewer feedback indicating limited evidence, the search strategy was comprehensively revised and rerun from inception (January 2015) through November 2025, using broader search terms across all 5 databases (Multimedia Appendix 1). Supplemental strategies included backward citation chasing of included articles and forward citation tracking of seminal papers.

Following duplicate removal via Covidence systematic review software (Veritas Health Innovation), 330 unique records underwent title or abstract screening by 2 independent reviewers (RZ and MS). The principal investigator (PI) (LH) served as the arbiter for conflicts. Calibration exercises on pilot samples demonstrated substantial interrater agreement (Cohen κ=0.78 for title or abstract screening, κ=0.82 for full-text review). Of the 330 records screened, 138 proceeded to full-text review. The same 2 independent reviewers conducted the full-text assessment and data extraction, with the PI resolving any disagreements. Full-text assessment excluded 130 studies primarily for the following reasons: clinical outcomes focus without implementation considerations (n=62, 47.7%), telehealth outside oncology without transferability (n=34, 26.2%), insufficient empirical or theoretical contribution (n=24, 18.5%), absence of rural/barrier focus (n=18, 13.8%), no AI or predictive analytics application (n=12, 9.2%), or inadequate methodological detail (n=8, 6.2%). Four studies met all inclusion criteria. Detailed screening procedures, interrater agreement calculations, and reasons for key exclusions are documented in Multimedia Appendix 2.

Data extraction used a standardized form to capture: study characteristics (design, setting, and population), tele-oncology implementation details (modality, clinical application), AI or predictive analytics applications (techniques, prediction targets, and performance metrics), implementation factors (barriers, facilitators, and strategies), and outcomes (implementation, clinical, and process measures). The extraction form, which was developed iteratively through pilot testing and team discussions, is provided in Multimedia Appendix 3.

Synthesis used narrative thematic analysis, which was appropriate for heterogeneous evidence that precluded meta-analysis. Following a modified framework synthesis, we organized findings into 3 prespecified themes aligned with the review objectives: (1) the current tele-oncology implementation landscape in rural and underserved settings, (2) potential AI-powered predictive analytics applications addressing implementation challenges, and (3) implementation considerations for AI systems themselves. Two independent reviewers (RZ and MS) coded the extracted data, with the principal investigator resolving disagreements and developing subthemes inductively through iterative discussion and consensus. Formal quality appraisal was not conducted, consistent with scoping review methodology [27]. As articulated in foundational frameworks, scoping reviews prioritize evidence mapping and gap identification over intervention effectiveness synthesis, which is the purview of systematic reviews requiring quality assessment.

## Results

**Figure 1.**
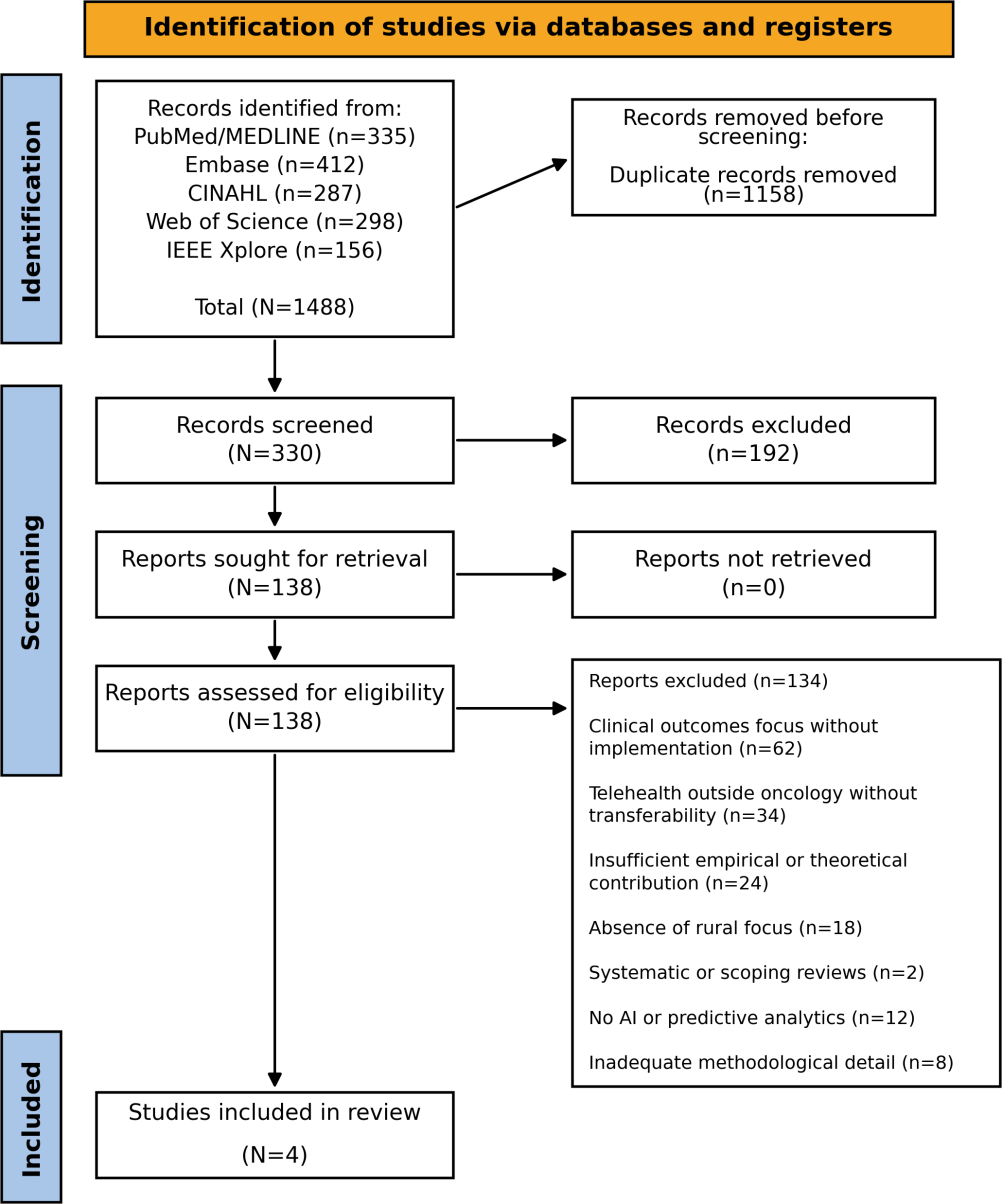
PRISMA (Preferred Reporting Items for Systematic Reviews and Meta-Analyses) flow diagram. AI: artificial intelligence; IEEE: Institute of Electrical and Electronics Engineers.

The systematic search yielded 330 unique records; 138 underwent full-text review, resulting in 4 included studies (Figure 1). This limited evidence base reflects the nascent intersection of AI-powered predictive analytics, tele-oncology implementation, and rural health equity—domains that have evolved largely in parallel rather than through integrated inquiry. The included studies comprised 2 pilot feasibility studies [32,33], 1 cross-sectional predictive study [34], and 1 platform development study [35], published between 2019 and 2025. Geographic concentration in high-resource settings (United States n=2, Greece n=1, and Singapore n=1) and limited oncology-specific focus underscore structural gaps in evidence generation for underserved populations.

**Figure 2.**
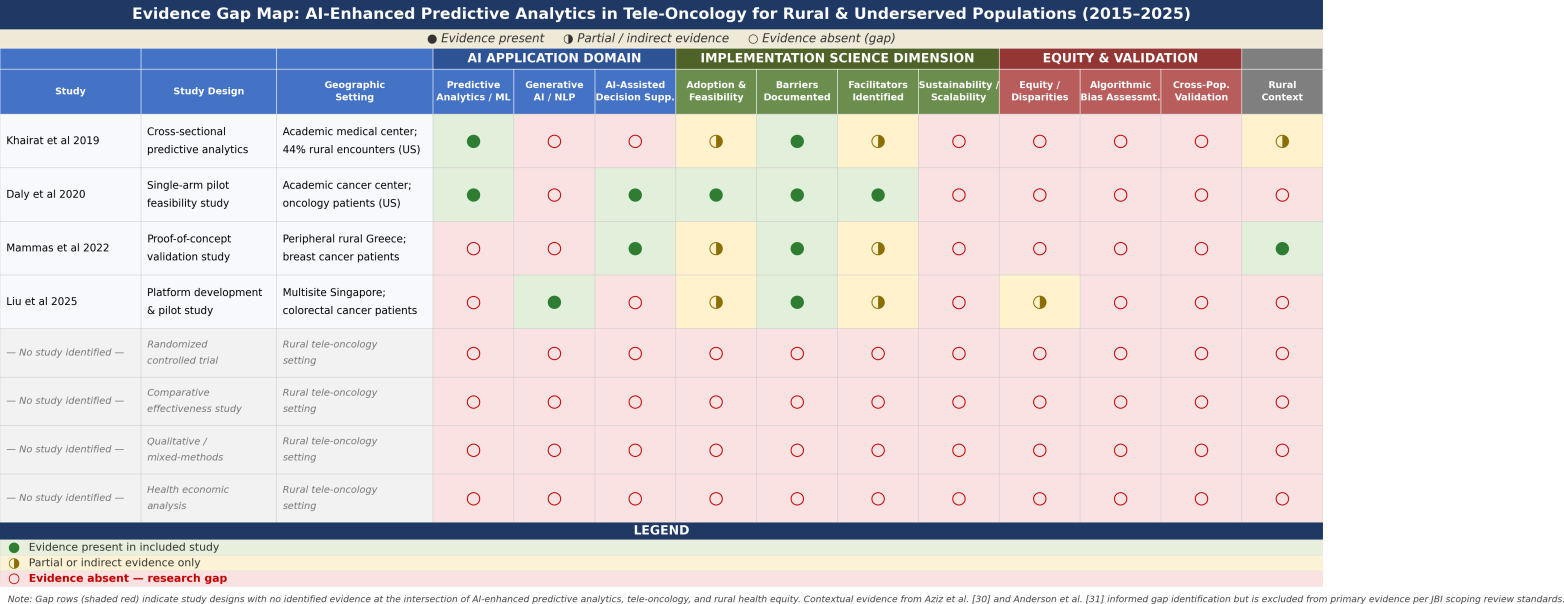
Evidence gap map [32-35]. ML: machine learning; NLP: natural language processing.

Table 1 presents study characteristics, including methodological approaches, populations, AI applications, and key limitations. Table 2 synthesizes implementation barriers across multilevel ecological domains. Table 3 maps AI-powered predictive analytics applications to specific tele-oncology implementation challenges, articulating translational pathways from current evidence to future research priorities. Figure 2 presents an evidence gap map illustrating the distribution of current evidence across AI application domains, implementation science dimensions, and equity and validation considerations.

**Table 1.** Characteristics of included studies (N=4).

Study	Design	Setting or population	AI^a^ or analytics application	Key implementation findings	Limitations
Daly et al [32], 2020	Single-arm pilot feasibility study	Academic cancer center regional site; n=100 high-risk patients initiating antineoplastic therapy	ML^b^ risk stratification model identifying high-risk patients for preventable acute care; daily PRO^c^ symptom surveillance with alert generation	29% enrollment rate; 56% daily response rate sustained 6 months; 93% generated severe alerts; 4 patient archetypes identified; 22% vs 39% acute care use (enrolled vs nonenrolled); provider volume threshold effects	No control group; single-site; resource-intensive (3 FTE^d^ for 100 patients); cost-effectiveness unknown
Mammas et al [33], 2022	Proof-of-concept validation study	Peripheral Greece (rural); n=40 patients with breast cancer (32 malignant, 8 benign)	AI-assisted remote multidisciplinary tumor boards integrating teleradiology, telepathology, telecytology with algorithmic interpretation	Mean accuracy: 94.1% surgical planning (95% CI: 85%‐99%), 96.8% chemotherapy, 96.7% hormonal therapy; addresses rural subspecialty access barrier	Small sample; breast cancer only; single health care system; generalizability unclear
Khairat et al [34], 2019	Cross-sectional predictive analytics	Academic medical center; n=1403 virtual urgent care encounters; 44% rural	Multinomial logistic regression predicting patient preferences for mHealth vs telemedicine based on demographics and chief concern	Sex (*P*=.004) and geographic setting (*P*<.001) significantly predicted modality choice; 86.2% prediction accuracy; rural patients 50% less likely to choose video; high satisfaction both modalities (91.1%, 89%)	Single-site; limited demographic diversity; selection bias (self-selected virtual care users)
Liu et al [35], 2025	Platform development and pilot study	This block should read single-site US (Emory University); n=40 (20 colorectal cancer patients + 20 caregivers); 55% disadvantaged (income ≤250% Federal Poverty Level, Medicaid, or uninsured)	Generative AI (ChatGPT, Pictory) creating accessible health literacy content at 6th-grade reading level with multimedia	8-week pilot: disadvantaged participants logged in 2.52 × more than nondisadvantaged; 3.98/5 satisfaction; addresses health literacy barrier disproportionately affecting rural or underserved	Short duration; small sample; single cancer type; generative AI accuracy or reliability concerns

a AI: artificial intelligence.

b ML: machine learning.

c PRO: patient-reported outcomes.

d FTE: full-time equivalent.

**Table 2.** Multilevel implementation barriers identified across the included studies (N=4).

Level	Barrier category	Specific barriers (studies reporting)
Patient	Technological access	Device ownership gaps [33,34]; broadband connectivity limitations, especially in rural areas [33,34]; inadequate infrastructure supporting telehealth access [33]
Patient	Digital literacy	Technological skill deficits [32,34,35]; low digital confidence or self-efficacy [33-35]; platform navigation difficulties [32,34]
Patient	Health literacy	Inability to interpret health information without support [35]; limited understanding of symptom significance [32,35]; educational barriers limiting self-management [35]
Patient	Psychosocial	Cancer-related distress limiting engagement [32]; preference for in-person care [33]; privacy/security concerns about data sharing [34]
Provider	Workflow integration	EMR^b^ integration complexities [32,33]; disrupted clinical workflows [32,34]; time requirements for reviewing AI-generated outputs [32,34]
Provider	Algorithmic trust	Algorithmic opacity hindering understanding [34]; uncertainty about appropriate reliance on predictive recommendations [32,34]
Provider	Clinical concerns	Medicolegal uncertainty regarding AI-supported care decisions [32]; inability to perform physical examination remotely [33]; care fragmentation concerns [32]
Provider	Training or support	Insufficient AI literacy among clinicians [32]; inadequate technical support infrastructure [32,34]; limited training on platform use [34]
Organizational	Technical infrastructure	EMR interoperability challenges [32-34]; data standardization deficits [34]; cybersecurity requirements [33]
Organizational	Human resources	Dedicated monitoring team requirements [32]; technical support staffing needs [32,34]; competing clinical priorities limiting engagement [32]
Organizational	Financial	High startup costs [32,34]; uncertain return on investment [32,34]; reimbursement ambiguities [34]; ongoing maintenance costs [32](
Organizational	Organizational culture	Resistance to change in clinical practice [32]; inadequate leadership support for tele-oncology implementation [32,34]; misaligned organizational incentives [34]
System	Infrastructure	Rural broadband inadequacies [33,34]; digital divide persistence in underserved regions [33,35]; geographic infrastructure limitations [33]
System	Regulatory or policy	Licensure barriers across state/national borders [32,34]; reimbursement policy limitations constraining sustainability [34]; data privacy or HIPAA^c^ compliance requirements [32]
System	Validation or standards	Lack of standardized validation frameworks for AI^a^ systems [32-34]; algorithmic bias in training data [34,35]

a AI: artificial intelligence.

b EMR: electronic medical record.

c HIPAA: Health Insurance Portability and Accountability Act.

**Table 3.** Translational pathways: mapping AI-powered predictive analytics to tele-oncology implementation challenges.

Implementation challenge	AI^a^ or predictive analytics application	Current evidence base	Research priorities
Low telehealth used among rural or underserved populations despite geographic barriers	Predictive models identifying patient subgroups likely to engage with specific telehealth modalities; personalized platform matching based on demographic and clinical characteristics; algorithmically informed outreach strategies	Khairat et al [34]: 86.2% accuracy predicting mHealth versus telemedicine preference based on sex, geographic setting, and chief concern; urban residents showed 101% increased odds of video selection vs rural (*P*<.001)	Validate modality prediction models in rural tele-oncology settings; develop actionable implementation protocols translating predictions into targeted outreach; examine sustainability of personalized matching approaches
Preventable acute care use during cancer treatment due to undetected symptom escalation	Risk stratification models identifying high-risk patients; real-time symptom surveillance with predictive escalation alerts; machine learning identifying patients at risk for acute care events	Daly et al [32]: ML^b^ risk stratification model targeting high-risk quartile; 93% of enrolled patients generated severe symptom alerts; preliminary data suggested 22% acute care usage vs 39% in nonenrolled high-risk controls	Conduct RCTs^c^ comparing AI-enhanced vs standard symptom monitoring; optimize alert threshold sensitivity or specificity; determine cost-effectiveness in rural cancer centers; validate across cancer types; examine long-term sustainability
Lack of subspecialty oncology expertise in rural facilities for multidisciplinary treatment planning	AI-assisted virtual tumor boards integrating imaging or pathology interpretation with algorithmic decision support; remote access to tumor board expertise via AI-augmented consultations	Mammas et al [33]: AI-assisted remote tumor boards achieved 94.1% accuracy for surgical planning (95% CI: 85%‐99%), 96.8% for chemotherapy recommendations, and 96.7% for hormonal therapy decisions in breast cancer cases (n=40)	Expand validation to multiple cancer types beyond breast cancer; validate across diverse health care systems and geographic regions; examine implementation feasibility in US rural cancer centers with limited technical infrastructure
Health literacy barriers limiting self-management capacity, disproportionately affecting rural/underserved populations	Generative AI creating personalized, accessible educational content tailored to individual literacy level, language preferences, and learning modality; AI-generated multimedia for symptom management and treatment adherence	Liu et al [35]: Generative AI platform creating 6th-grade reading level content with multimedia enhancement; disadvantaged participants (income <$50,000 or education ≤ high school) demonstrated 2.52-fold higher engagement compared to nondisadvantaged counterparts	Validate generative AI accuracy and reliability in oncology contexts; test across cancer types and treatment modalities; examine long-term outcomes on self-management and treatment adherence; assess equity in content generation
Inadequate resources for proactive identification and outreach to patients experiencing implementation barriers	Predictive models identifying patients at elevated risk for technology access barriers, digital literacy deficits, and other implementation obstacles using SDOH^d^ data; automated risk flagging enabling targeted interventions	Theoretical frameworks emphasized [32,34] but no empirical validation in tele-oncology contexts	Develop and validate barrier prediction models integrating SDOH data; design targeted intervention protocols addressing identified barriers; test implementation in rural cancer centers; examine cost-effectiveness
Workflow integration challenges and provider resistance limiting adoption of tele-oncology platforms	AI-assisted clinical decision support reducing cognitive burden; automated clinical documentation generating summaries from encounters; intelligent prioritization of high-risk patients; systems learning from provider feedback to improve usability	Frameworks emphasize sociotechnical integration [32,34] with emphasis on perceived usefulness and ease of use, but limited empirical testing of specific AI features addressing workflow pain points	Develop AI tools targeting specific workflow pain points identified through provider ethnography; conduct rigorous usability testing; measure time savings and clinician satisfaction; examine adoption and fidelity in rural settings
Algorithmic bias in AI systems potentially exacerbating existing health disparities in rural cancer care	Fairness-aware machine learning approaches; bias detection algorithms monitoring performance across demographic groups; continuous equity monitoring of disparate impact; diverse training data representation; transparent performance reporting disaggregated by population	Theoretical emphasis on equity monitoring [32,34] but minimal empirical application in tele-oncology	Develop equity-focused validation frameworks requiring intersectional analyses; mandate diverse, representative training datasets; implement real-time disparities monitoring; publish disaggregated performance metrics by race, ethnicity, SES^f^, geographic location; establish accountability mechanisms

a AI: artificial intelligence.

b ML: machine learning.

c RCT: randomized controlled trial.

d SDOH: Social Determinants of Health.

e SES: socioeconomic status.

Khairat et al [34] demonstrated that patient characteristics predict telehealth modality preferences with sufficient accuracy (86.2%) to inform personalized implementation strategies. Analyzing 1403 virtual care encounters, multinomial logistic regression revealed sex (*P*=.004) and geographic setting (*P*<.001) as primary determinants: male patients exhibited 66% increased odds of video telemedicine selection versus female patients, while urban residents showed 101% increased odds compared to their rural counterparts. Chief medical concern independently predicted modality choice (*P*<.001), with privacy-sensitive conditions (eg, urinary tract infections and gynecological complaints) associating with telephone preference and visual assessments (eg, dermatological concerns and conjunctivitis) predicting video selection. High satisfaction persisted across modalities (91.1% mobile health and 89% telemedicine), suggesting that offering choice and algorithmically matching patients to appropriate platforms enhances acceptability without compromising outcomes.

These findings illuminate how predictive analytics can address the implementation paradox, wherein individuals experiencing the greatest geographic barriers demonstrate the lowest telehealth usage. By identifying patient subgroups likely to engage with specific modalities, health care systems can proactively tailor outreach, technology support, and platform recommendations—moving beyond one-size-fits-all implementation toward precision approaches responsive to intersecting social determinants.

Three studies examined AI applications in oncology remote monitoring, revealing feasibility alongside persistent implementation challenges. Daly et al [32] integrated machine learning risk stratification with digital symptom surveillance for 100 high-risk patients initiating antineoplastic therapy. The predictive model prospectively identified patients at elevated risk for preventable acute care usage, enabling targeted enrollment. Daily patient-reported outcome assessments achieved a 56% sustained response rate over 6 months, with 93% of enrolled patients generating severe symptom alerts requiring clinical intervention. Qualitative analysis identified 4 patient engagement archetypes—Promise Keeper, Data Tracker, Unengaged, and Overwhelmed—each characterized by distinct motivational drivers and implementation needs, underscoring heterogeneity in technology acceptance even among self-selected participants.

Preliminary acute care data suggested a potential benefit (22% usage among enrolled patients vs 39% among nonenrolled high-risk controls), though the absence of a randomized design precludes causal inference. Critical implementation barriers emerged: providers with low patient volumes perceived information overload and care fragmentation, while those with adequate panel penetration valued the intervention, suggesting threshold effects for workflow integration. Organizational requirements—dedicated nursing team (2 registered nurses and 1 nurse practitioner for 100 patients), custom electronic medical record (EMR) integration, and 12-hour daily coverage—raise scalability concerns for rural facilities with constrained resources.

Mammas et al [33] demonstrated the technical feasibility of AI-assisted remote multidisciplinary tumor boards for breast cancer in peripheral Greece, achieving mean accuracy of 94.1% for surgical planning, 96.8% for chemotherapy, and 96.7% for hormonal therapy (95% CI 85%–99% for all 3 estimates), across 40 cases. This model addresses a fundamental rural implementation barrier—the absence of local subspecialty expertise—by leveraging AI to augment telehealth consultations. However, generalizability beyond breast cancer and validation in diverse health care contexts remain unexplored.

Liu et al [35] used generative AI to create accessible health literacy content for colorectal cancer self-management, targeting disadvantaged populations at a single US academic medical center. The platform automatically generated 6th-grade reading level materials with multimedia enhancement. Disadvantaged participants (defined as income ≤250% of the Federal Poverty Level, Medicaid enrollment, or uninsured status) demonstrated 2.52-fold higher platform engagement than nondisadvantaged counterparts, suggesting particular value for populations experiencing compounding socioeconomic marginalization. This application illustrates how AI can address specific implementation barriers, such as inadequate health literacy, that disproportionately constrain rural and underserved community engagement with tele-oncology.

Cross-study synthesis reveals fundamental evidence gaps. No included studies directly examined AI-powered predictive analytics for identifying or mitigating tele-oncology implementation barriers in rural settings—the core focus of this review. Validation of predictive models across diverse populations, health care systems, and geographic contexts remains limited, with most studies conducted in single academic medical centers serving nonrepresentative populations. Translation of AI predictions into actionable implementation strategies has received minimal empirical examination. Cost-effectiveness analyses comparing AI-enhanced approaches versus standard approaches were notably absent. Longitudinal research examining sustainability, adaptation, and the impact on disparities beyond pilot phases would strengthen the evidence for scaling.

Table 2 synthesizes implementation barriers across patient, provider, organizational, and system levels. Patient-level barriers encompassed technological literacy deficits, device access limitations, broadband connectivity constraints, and digital confidence gaps—challenges disproportionately affecting older adults, individuals from lower socioeconomic populations, and people living in rural communities. Provider barriers included workflow integration complexities, algorithmic opacity concerns, time requirements for reviewing AI-generated outputs, and medicolegal uncertainty regarding appropriate reliance on algorithmic recommendations. Organizational barriers spanned technical infrastructure (EMR integration and data interoperability), staffing resources (dedicated monitoring teams and technical support), and financial sustainability (startup costs, ongoing maintenance, and reimbursement uncertainties). System-level barriers encompassed broadband infrastructure inadequacies in rural areas, regulatory ambiguities, data privacy requirements, and the absence of standardized validation frameworks.

**Figure 3.**
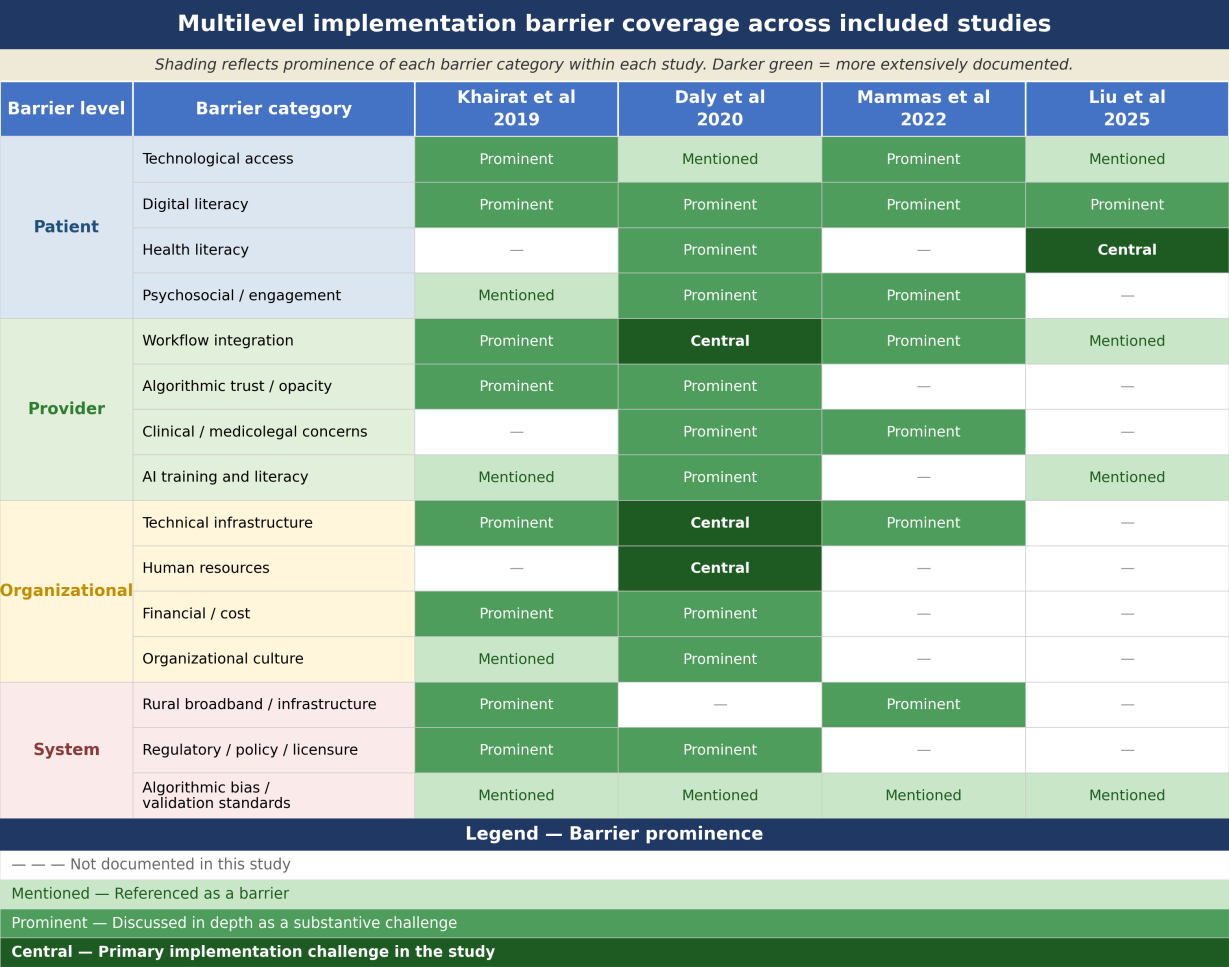
Barrier heat map [32-35]. AI: artificial intelligence.

Figure 3 illustrates the relative prominence of each barrier category within each included study, highlighting areas where challenges to implementation challenges were most extensively documented.

Facilitators enabling implementation success emerged consistently (Table 3): strong clinician champions actively reinforcing program value with patients, a responsive technical support infrastructure, demonstrable clinical benefits driving stakeholder buy-in, and sustained organizational commitment beyond pilot funding. The evidence suggests that AI implementation success depends less on technological sophistication per se than on systematic attention to sociotechnical integration—aligning AI capabilities with clinical workflows, organizational contexts, and patient needs through iterative, stakeholder-engaged development and deployment processes.

## Discussion

### Principal Findings

This scoping review sought to (1) characterize current applications of AI and predictive analytics in telehealth and digital oncology care; (2) examine empirical evidence on implementation factors, barriers, and facilitators; and (3) identify research gaps and future directions for optimizing tele-oncology implementation in rural contexts. Across all 3 objectives, the overarching finding is one of substantial evidence scarcity. Of 330 unique records screened, only 4 primary studies [32-35] met inclusion criteria, none of which directly tested the integration of AI-enhanced predictive analytics within a rural tele-oncology implementation context. The included studies offered selective proof-of-concept across discrete AI applications (modality preference prediction, remote symptom monitoring, AI-assisted multidisciplinary tumor boards, and generative health literacy tools): yet each was conducted in a well-resourced or academic setting, with limited applicability to the rural contexts that are the focus of this review. Multilevel implementation barriers were consistently documented across all 4 studies, and no study examined algorithmic bias, cross-population validation, or equity-related harms. These findings suggest that the field remains at an early, exploratory stage, and that current evidence is insufficient to support practice recommendations or policy guidance for rural tele-oncology implementation.

### Technical Feasibility and the Gap to Implementation Readiness

The included studies collectively suggest that AI-based tools may, under specific conditions, address discrete implementation challenges in oncology care delivery. Khairat et al [34] found that patient demographic and clinical characteristics were sufficiently predictive of telehealth modality preference to potentially inform personalized outreach strategies, a finding with plausible relevance for rural populations, who exhibit lower rates of video-based telehealth adoption in the broader literature [14,17]. Daly et al [32] demonstrated the operational feasibility of machine learning risk stratification integrated with digital symptom surveillance, with preliminary signals suggesting reduced acute care use among enrolled patients, though the absence of a control group precludes causal inference. Mammas et al [33] reported the technical accuracy of AI-assisted remote tumor boards for breast cancer management in rural Greece, offering a possible model for extending subspecialty access to geographically isolated facilities [15,16]. Liu et al [35] showed that generative AI can produce health literacy materials that may engage socioeconomically disadvantaged populations more effectively than standard content, though this finding was observed in a single cancer type in Singapore, and its generalizability to rural Western contexts warrants caution.

However, this provisional feasibility evidence coexists with documented implementation fragility. Daly et al [32] observed organizational threshold effects, whereby the remote monitoring intervention functioned adequately when provider panel penetration was sufficient, but generated information overload and care fragmentation concerns under lower-volume conditions—conditions more representative of rural cancer facilities [14]. The intervention’s staffing requirements raise additional scalability questions for resource-constrained settings [32]. These tensions are echoed in the broader contextual literature. Aziz et al [30], synthesizing 11 studies of AI-driven remote patient monitoring in oncology, concluded that available evidence remains insufficient to establish superiority over traditional care delivery, identifying consistent barriers including technical infrastructure requirements, gaps in literacy among patients and providers, and workflow integration complexities. The fact that this conclusion emerged from studies largely conducted in well-resourced academic environments further underscores the depth of the evidence gap for rural contexts. Anderson et al [31] noted that only 26% of the telehealth predictive analytics studies they reviewed focused on predictive systems, with a notable absence of resource optimization models applicable to rural or underresourced implementation contexts. This suggests that the orientation of existing research may be poorly matched to the implementation challenges that rural settings face [18,19,31].

### Persistence of Multilevel Implementation Barriers

A consistent pattern across the primary studies and contextual review literature is that implementation barriers operate simultaneously across patient, provider, organizational, and system levels, and that technological sophistication does not, in itself, resolve barriers operating at other levels [7,14,18]. At the patient level, device access limitations, broadband connectivity gaps, digital literacy deficits, and health literacy constraints appear to intersect in compounding ways, with particular salience for older adults, individuals with lower incomes, and people living in geographically isolated areas [13,14,17]. Khairat et al [34] found that people living in rural areas were significantly less likely to select video-based telehealth even when it was available, suggesting that geographic context independently shapes technology engagement beyond questions of mere access. At the provider level, algorithmic opacity (clinicians’ difficulty in interpreting and appropriately acting on AI-generated outputs) emerges as a recurrent concern across studies [32,34], and is identified by Aziz et al [30] as a barrier to adoption of AI-enhanced monitoring across multiple health care systems. Reddy [24] similarly argues that translating AI tools into clinical practice requires governance structures and workflow integration processes that most current deployment efforts have not adequately addressed.

Organizational and system-level barriers may be especially consequential for rural settings. The dedicated staffing, EMR integration, and sustained technical support required by the Daly et al [32] intervention represent structural prerequisites that are unlikely to be available in many rural facilities [14,15]. Anderson et al [31] noted the absence of resource optimization models in the telehealth analytics literature, suggesting that the field has yet to systematically engage with how AI tools might be deployed sustainably under resource constraints. At the system level, rural broadband inadequacies, cross-border licensure ambiguities, and reimbursement policy limitations add further layers of complexity compounding facility-level constraints and potentially limiting the transferability of findings from academic settings [13,14,19].

### Equity Considerations and Gaps in the Evidence Base

A notable gap across all 4 primary studies is the absence of equity-centered analysis. None of the included studies explicitly examined algorithmic bias, conducted cross-population validation of AI system performance across racial, socioeconomic, or geographic subgroups, or assessed potential harms associated with AI deployment in underserved populations. This gap is particularly concerning given documented evidence that AI systems trained predominantly on data from majority or well-resourced populations may perform differentially—and potentially harmfully—when applied to underrepresented communities [5,18,19]. Individuals with cancer living in rural and lower-income areas, who are already subject to compounding health care disadvantages, including later-stage diagnoses and poorer survival outcomes [15,16], may be disproportionately exposed to such risks.

Aziz et al [30] explicitly identified insufficient attention to equity impacts as a limitation of the AI-enhanced remote monitoring literature, observing that reviewed studies rarely examined whether benefits were distributed equitably across patient subgroups or whether the implementation burden fell disproportionately on already-disadvantaged populations. Anderson et al [31] similarly found that the vast majority of telehealth predictive analytics studies were conducted in single academic medical centers serving nonrepresentative populations, with minimal rural implementation focus and no systematic reporting of outcomes disaggregated by socioeconomic or demographic characteristics. Taken together, these observations suggest that populations with the greatest potential need for equitable tele-oncology implementation may be systematically underrepresented in the research base intended to inform it [5,17,18].

The implications of this gap extend beyond research design. Maw et al [18] argue that achieving equitable AI implementation in health care likely requires pragmatic implementation science approaches that explicitly center population diversity, contextual adaptation, and community engagement—an orientation that appears largely absent from the current evidence base. Trinkley et al [19] further caution that AI applications in implementation science carry the risk of perpetuating or amplifying existing disparities if equity considerations are not prospectively embedded in system design, validation protocols, and ongoing monitoring frameworks. Without deliberate attention to whose data informs AI models and whose outcomes are measured, there is a meaningful risk that AI-enhanced tele-oncology tools could widen rather than narrow existing cancer care disparities for rural and underserved populations [5,18,19].

### Implications for Future Research

Table 4 summarizes the identified research priorities, mapped to the gap type, supporting evidence, and ratings of urgency, feasibility, and equity relevance.

**Table 4.** Research priorities matrix.

Research priority	Gao type	Primary evidence (this review)	Contextual support	Urgency	Near-term feasibility	Equity relevance
Comparative effectiveness studies measuring implementation outcomes (adoption, fidelity, penetration, and sustainability) in authentic rural health care settings, with longitudinal follow-up beyond initial pilot phases	Methodological gap	Daly et al [32], Mammas et al [33]	[7,25,26]	High	Low	High
Cross-population validation of AI^a^ systems, with performance disaggregated by rural versus urban status, race or ethnicity, socioeconomic position, and digital literacy level	Equity or validation gap	All 4 studies (absent in each)	Aziz et al [30], Anderson et al [31]	High	Medium	High
Research specifying translation mechanisms linking AI predictions to actionable implementation strategies, examining how predictions are acted upon by clinicians and organizations	Implementation science gap	Khairat et al [34], Daly et al [32]	Trinkley et al [ 19]; [7,19,24]	High	Medium	Medium
Health economic analyzes comparing AI-enhanced versus standard implementation approaches in rural settings, informing infrastructure investment, workforce, and reimbursement decisions	Economic evidence gap	Daly et al [32], Khairat et al [34]	Absent from current literature	Medium	Medium	Medium
Qualitative and mixed methods research on sociotechnical integration in resource-constrained settings, and development or testing of implementation science–informed governance frameworks	Mechanistic or governance gap	Daly et al [32]	Reddy [24]; [12,24]	Medium	High	Medium

a AI: artificial intelligence.

The gaps identified through this review point to several interrelated priorities for future investigation. Perhaps most foundationally, the field would benefit from comparative effectiveness studies conducted in authentic rural health care settings (rather than academic medical centers) that measure implementation science outcomes, including adoption, fidelity, penetration, and sustainability, alongside clinical end points [7,25,26]. Scoping reviews such as this one can identify where evidence is absent, but only prospectively designed trials and implementation studies can determine whether AI-enhanced approaches offer meaningful advantages over standard care under realistic rural conditions. Longitudinal designs capable of capturing sustainability beyond initial pilot phases are likely necessary, given that existing feasibility studies were of limited duration and scope and cannot speak to whether observed benefits persist once dedicated research support is withdrawn [32,33].

Cross-population validation of AI systems represents a further priority of considerable urgency, with performance reporting disaggregated by rural versus urban status, race and ethnicity, socioeconomic position, and digital literacy level. Both Aziz et al [30] and Anderson et al [31] identify this as largely unaddressed in current literature, and the absence of such validation means that the equity implications of deploying existing AI tools in rural oncology contexts remain genuinely unknown. Relatedly, research that specifies the translation mechanisms linking AI predictions to actionable implementation strategies would address a theoretical gap articulated by Trinkley et al [19] but not yet empirically examined in rural oncology contexts. Understanding not just whether AI can predict an outcome, but how those predictions are acted upon by clinicians and organizations, and under what contextual conditions, is essential for implementation science to move beyond feasibility demonstration [7,19,24].

Health economic analyses comparing AI-enhanced and standard implementation approaches in rural settings are notably absent from the current evidence base [32,34], yet they are likely to be important for policy decisions regarding infrastructure investment, workforce development, and reimbursement, ultimately determining whether AI-enhanced tele-oncology becomes accessible to rural populations at scale. Finally, qualitative and mixed-methods research examining sociotechnical integration in resource-constrained settings would provide mechanistic insight into the organizational and cultural factors enabling or constraining implementation success—insight that quantitative approaches alone cannot offer [12,24]. Reddy [24] argues that implementation science-informed governance frameworks are a prerequisite for responsible AI deployment in health care; developing and testing such frameworks in rural oncology contexts represents a meaningful and tractable research contribution.

### Limitations

Several limitations of this review should be considered when interpreting its findings. The small number of included primary studies (n=4), their heterogeneous designs, and their concentration in single academic or high-resource sites preclude comparative synthesis and limit generalizability. Geographic concentration in the United States, Greece, and Singapore means that findings may not reflect implementation contexts in low-income and middle-income countries or in rural settings with more severe infrastructure limitations. The English-language restriction may have introduced systematic bias by excluding relevant non-Anglophone literature. Formal quality appraisal was not conducted, consistent with scoping review methodology [26,27], which means the internal validity of individual study findings remains unassessed. The rapid pace of AI development also means that applications currently under development or reported in gray literature may not yet be captured in the peer-reviewed evidence base, and implementation-focused research characteristically lags behind technology development [19,24]. Finally, while Aziz et al [30] and Anderson et al [31] are used as contextual evidence throughout this discussion, their conclusions are themselves subject to the limitations of the primary studies they synthesized, including heterogeneous quality and limited rural-specific analysis.

### Conclusions

This scoping review suggests that the potential of AI-enhanced predictive analytics to improve tele-oncology implementation in rural and underserved settings, while theoretically plausible, remains empirically underdeveloped. The 4 included primary studies offer isolated proof-of-concept evidence but collectively illustrate a pattern in which technological capability may outpace implementation readiness, and in which the structural constraints most characteristic of rural settings receive comparatively limited research attention. The broader contextual literature reinforces this assessment: Aziz et al [30] found that AI-enhanced remote monitoring in oncology has not yet demonstrated clear superiority over standard care, and Anderson et al [31] observed that predictive analytics research in telehealth has largely not addressed the resource optimization and implementation science questions most relevant to populations in underserved areas.

The systematic underrepresentation of rural, low-income, and racially diverse populations in AI and digital health research is not merely an empirical gap—it may reflect and perpetuate structural inequities in knowledge production that have downstream consequences for health equity [5,18]. Deploying AI tools with unexamined performance characteristics in contexts where patients already experience compounding disadvantages carries meaningful risks that the field has not yet adequately engaged with [15,16,19]. Reddy [24] and Trinkley et al [19] both suggest that responsible AI implementation in healthcare requires equity monitoring, community engagement, and governance frameworks that remain underdeveloped in current practice. Until such frameworks are established and empirically validated in rural oncology contexts, the risk that AI-enhanced tele-oncology reproduces rather than reduces existing disparities warrants serious consideration.

Advancing this field equitably will likely require a reorientation of the research agenda toward community-engaged, implementation science-informed study designs conducted in authentic rural contexts, with explicit attention to equity, sustainability, and population diversity [7,18,25,26]. Rural communities should ideally participate as collaborators in the design and governance of AI tools intended to serve them, ensuring that research questions are grounded in authentic implementation challenges [18,19]. Funding priorities, editorial standards, and implementation science frameworks that incentivize disaggregated outcome reporting, long-term sustainability research, and equity-centered validation may support this reorientation [5,7,26]. Only through such an approach can the theoretical promise of AI-enhanced tele-oncology be meaningfully evaluated and responsibly translated for the populations who may stand to benefit most.

## Supplementary material

10.2196/78005 Multimedia Appendix 1 Search strategy.

10.2196/78005 Multimedia Appendix 2 Inclusion and exclusion criteria.

10.2196/78005 Multimedia Appendix 3 Data extraction form.

10.2196/78005 Checklist 1 PRISMA-ScR checklist.

10.2196/78005 Checklist 2 PRISMA-S checklist.

## Abbreviations

AI: artificial intelligence
EMR: electronic health record
PRISMA-S: Preferred Reporting Items for Systematic Reviews and Meta-Analyses Literature Search Extension
PRISMA-SCR: Preferred Reporting Items for Systematic Reviews and Meta-Analyses Extension for Scoping Reviews
