# Supplementary material for: AI-Enhanced Predictive Analytics to Optimize Tele-Oncology Implementation in Rural Settings: Scoping Review
Source: JMIR Cancer. 2026 Jul 16;12:e78005. doi: 10.2196/78005 (PMC13374798; doi:10.2196/78005)
Supplement: Multimedia Appendix 1 [file cancer-v12-e78005-s001.docx]

**Supplementary File 2: Search Strategy**

# Search Overview

**Databases:** PubMed/MEDLINE, Embase, CINAHL Complete, Web of Science Core Collection, IEEE Xplore Digital Library

**Search Dates:** Initial search April 9, 2025; Expanded search November 21-24, 2025

**Date Range:** January 1, 2015 – November 2025

**Language:** English only

# Table 1. Core Search Concepts and Terms

| **Concept 1: Tele-oncology/ Telehealth** | **Concept 2: Cancer/ Oncology** | **Concept 3: Rural/ Implementation** | **Concept 4: AI/Predictive Analytics** |
| --- | --- | --- | --- |
| telehealth, tele-health, telemedicine, tele-medicine, virtual care, virtual health, digital health, ehealth, e-health, mhealth, m-health, mobile health, remote care, remote healthcare, distance medicine, video visit*, video consultation*, video conferenc*, videotelemedicine, teleconsultation*, tele-consultation*, remote consultation*, virtual visit*, virtual consultation*, online consultation*, teleoncology, tele-oncology, virtual oncology, remote oncology, virtual tumor board*, remote tumor board*, remote monitoring, remote patient monitoring, telemonitoring, tele-monitoring, home monitoring | cancer*, oncolog*, neoplasm*, tumor*, tumour*, malignancy, malignancies, carcinoma*, sarcoma*, lymphoma*, leukemia, leukaemia, melanoma* | rural, rural area*, rural population*, rural community, rural communities, rural setting*, frontier, remote area*, remote region*, remote location*, remote population*, underserved area*, medically underserved, health professional shortage area*, HPSA, geographically isolated, geographic isolation, geographic barrier*, healthcare access, health care access, access to care, access barrier*, health disparity, health disparities, healthcare disparity, healthcare disparities, health equity, inequit*, implementation, implement*, adoption, adopt*, uptake, utilization, utilisation, barrier*, obstacle*, challenge*, facilitat*, enabler*, technology adoption, digital adoption, digital divide, broadband access, internet access, connectivity, digital literacy, health literacy, ehealth literacy, implementation strategy, implementation strategies, implementation outcome*, implementation barrier*, dissemination, diffusion, sustainment, sustainability, scale-up, scaling, feasibility, acceptability, appropriateness, healthcare delivery, health care delivery, service delivery, care delivery, delivery model*, integrated care, care coordination, hub-and-spoke | artificial intelligence, AI, machine learning, ML, deep learning, neural network*, convolutional neural network*, CNN, recurrent neural network*, RNN, LSTM, supervised learning, unsupervised learning, ensemble learning, random forest*, decision tree*, gradient boosting, XGBoost, support vector machine*, SVM, predictive model*, predictive modeling, predictive modelling, predictive analytics, predictive algorithm*, prediction model*, risk prediction, risk stratification, risk score*, prognostic model*, forecast*, decision support, clinical decision support, decision support system*, CDSS, computer-aided decision, computerized decision support, intelligent system*, expert system*, data mining, data science, big data, predictive informatics, health informatics, medical informatics, computational intelligence, statistical learning, natural language processing, NLP, text mining, text analysis, sentiment analysis, topic modeling, algorithm*, computational model*, mathematical model*, optimization, optimisation, resource allocation, capacity planning, workflow optimization |

*Note: All four concepts were combined using Boolean AND logic. Within each concept, terms were combined using OR. The asterisk (*) represents truncation for word variations.*

# Table 2. Database-Specific Search Strategies

| **Database** | **Final Search Strategy** | **Results** |
| --- | --- | --- |
| **Ovid MEDLINE(R) <1946 to Nov 21, 2025>** | **MeSH Terms + Keywords:**  Telemedicine: exp telemedicine/ OR exp telehealth/ OR keywords in ti,ab  Cancer: exp neoplasm/ OR exp oncology/ OR keywords in ti,ab  Rural/Implementation: exp rural health care/ OR exp rural area/ OR exp rural population/ OR exp medically underserved area/ OR exp health care access/ OR exp implementation/ OR exp technology assessment/ OR exp diffusion of innovation/ OR keywords in ti,ab  AI/Analytics: exp artificial intelligence/ OR exp machine learning/ OR exp deep learning/ OR exp neural network/ OR exp decision support system/ OR keywords in ti,ab  **Boolean: (Tele-oncology) AND (Cancer) AND (Rural/Implementation) AND (AI/Analytics)**  Limits: English, 2015-2025 | **335** |
| **Embase (Elsevier) <1947 to Nov 21, 2025>** | **Emtree Terms + Keywords:**  Telemedicine: 'telemedicine'/exp OR 'telehealth'/exp OR 'teleconsultation'/exp OR 'telemonitoring'/exp OR keywords in ti,ab  Cancer: 'neoplasm'/exp OR 'oncology'/exp OR 'cancer'/exp OR keywords in ti,ab  Rural/Implementation: 'rural health care'/exp OR 'rural area'/exp OR 'rural population'/exp OR 'medically underserved area'/exp OR 'health care access'/exp OR 'implementation'/exp OR keywords in ti,ab  AI/Analytics: 'artificial intelligence'/exp OR 'machine learning'/exp OR 'deep learning'/exp OR 'neural network'/exp OR 'decision support system'/exp OR keywords in ti,ab  **Boolean: (Tele-oncology) AND (Cancer) AND (Rural/Implementation) AND (AI/Analytics)**  Limits: English, Article or Review, 2015-2025 | **412** |
| **CINAHL Complete (EBSCOhost) <1937 to Nov 21, 2025>** | **CINAHL Headings + Keywords:**  Telemedicine: (MH "Telemedicine+") OR (MH "Telehealth+") OR keywords in TI/AB  Cancer: (MH "Neoplasms+") OR (MH "Oncology+") OR keywords in TI/AB  Rural/Implementation: (MH "Rural Health+") OR (MH "Rural Population+") OR (MH "Medically Underserved Area+") OR (MH "Health Services Accessibility+") OR (MH "Diffusion of Innovation+") OR keywords in TI/AB  AI/Analytics: (MH "Artificial Intelligence+") OR (MH "Machine Learning+") OR (MH "Deep Learning+") OR (MH "Neural Networks+") OR (MH "Decision Support Systems, Clinical+") OR keywords in TI/AB  **Boolean: (Tele-oncology) AND (Cancer) AND (Rural/Implementation) AND (AI/Analytics)**  Limits: English, Academic Journals, 2015-2025 | **287** |
| **Web of Science Core Collection (Clarivate) <2015 to Nov 21, 2025>** | **Topic Search (TS) - searches title, abstract, author keywords, Keywords Plus:**  TS=(all Concept 1 terms) AND TS=(all Concept 2 terms) AND TS=(all Concept 3 terms) AND TS=(all Concept 4 terms)  Refined by: English language, 2015-2025  Indexes: SCI-EXPANDED, SSCI, A&HCI, CPCI-S, CPCI-SSH, ESCI | **298** |
| **IEEE Xplore Digital Library <2015 to Nov 21, 2025>** | **Abstract Field Search:**  ("Abstract":all Concept 1 terms) AND ("Abstract":all Concept 2 terms) AND ("Abstract":all Concept 3 terms) AND ("Abstract":all Concept 4 terms)  Filters: English, Conferences/Journals/Early Access, 2015-2025 | **156** |

**Total Records Retrieved: 1,488**

**Unique Records After Deduplication: 330**

# Table 3. Sample MEDLINE Search Strategy (Ovid Syntax)

| **#** | **Query** | **Results** |
| --- | --- | --- |
| 1 | exp telemedicine/ or exp telehealth/ | 55,308 |
| 6 | 1 or 2 or 3 or 4 or 5 | 79,806 |
| 7 | exp neoplasm/ or exp oncology/ or exp cancer/ | 4,180,013 |
| 9 | 7 or 8 | 4,970,713 |
| 10 | 6 and 9 | 5,794 |
| 20 | 11 or 12 or 13 or 14 or 15 or 16 or 17 or 18 or 19 | 3,954,993 |
| 29 | 21 or 22 or 23 or 24 or 25 or 26 or 27 or 28 | 1,831,380 |
| 30 | 10 and 20 and 29 | 401 |
| **31** | **limit 30 to (english language and yr="2015-Current")** | **335** |

*Notes: MeSH terms were exploded (exp) to include all narrower terms. .ti,ab. = title and abstract fields. $ = truncation symbol in Ovid. Similar strategies were adapted for other databases using their specific syntax and controlled vocabularies. See full search strategy documentation for complete line-by-line searches for lines 2-5, 8, 11-19, and 21-28.*

# Supplemental Search Methods

**Citation Searching:**

• Backward citation chasing of included studies' reference lists • Forward citation tracking of seminal papers using Web of Science and Google Scholar

**Gray Literature:**

Not systematically searched given focus on peer-reviewed literature

# Search Strategy Development and Validation

**Development:** Search strategies were developed in consultation with a health sciences librarian and followed PRISMA-S guidelines

**Validation:** Key article retrieval test confirmed the strategy captured 4 known relevant studies identified during preliminary scoping

**Iteration:** Following initial April 2025 search, November 2025 expanded search included broader implementation science terms, additional AI technique variations, and more inclusive rural/access terminology to maximize sensitivity
