# Supplementary material for: AI-Enhanced Predictive Analytics to Optimize Tele-Oncology Implementation in Rural Settings: Scoping Review
Source: JMIR Cancer. 2026 Jul 16;12:e78005. doi: 10.2196/78005 (PMC13374798; doi:10.2196/78005)
Supplement: Multimedia Appendix 2 [file cancer-v12-e78005-s002.docx]

**Supplementary File 3: Complete Inclusion and Exclusion Criteria**

# Table 1. Population and Setting Criteria

| **Criterion** | **Operational Definition** | **Examples of Inclusion** | **Rationale** |
| --- | --- | --- | --- |
| **Rural/Underserved Populations (Priority but not required)** | Studies examining healthcare delivery in rural areas, remote regions, geographically isolated populations, medically underserved areas, health professional shortage areas, or populations facing significant geographic/access barriers. Rurality defined using author definitions including RUCA codes, population density, geographic distance to care, or self-identified rural status. | • Studies in RUCA-defined rural counties • Telehealth connecting urban cancer centers with remote clinics • Implementation in health professional shortage areas • Studies addressing geographic isolation barriers • Hub-and-spoke models serving rural populations | Rural focus is a priority given implementation challenges and health disparities. However, given limited evidence, we included studies from any setting with transferable implementation insights, allowing broader evidence synthesis while maintaining ability to identify rural-specific gaps. |
| **Cancer/Oncology Focus (Priority but not required)** | Studies examining oncology care delivery, cancer patients, oncology providers, or cancer treatment settings. Included all cancer types, stages, and treatment phases. Also included non-oncology telehealth/AI studies with clearly transferable implementation insights for oncology contexts. | • Tele-oncology consultations for any cancer type • Remote monitoring during chemotherapy • Virtual tumor boards • AI prediction models for cancer outcomes • General telehealth implementation studies with oncology-relevant barriers | Oncology-specific evidence is ideal but limited. Including general telehealth/AI studies allows examination of implementation patterns and barriers applicable to complex chronic disease management, which characterizes cancer care. |

# Table 2. Intervention and Technology Criteria

| **Criterion** | **Operational Definition** | **Examples of Inclusion** | **Rationale** |
| --- | --- | --- | --- |
| **Telehealth/Tele-oncology Modalities** | Studies examining any synchronous or asynchronous telehealth modality including video consultations, telephone encounters, remote patient monitoring, store-and-forward telemedicine, mobile health applications, virtual tumor boards, or hybrid delivery models combining telehealth with in-person care. | • Synchronous video consultations • Asynchronous teleconsultation platforms • Remote symptom monitoring via apps • Store-and-forward teleradiology • Hybrid models (telehealth + in-person) | Broad inclusion of telehealth modalities captures diverse implementation approaches and technological solutions applicable to tele-oncology contexts, recognizing that optimal delivery models may vary by setting and population. |
| **AI/Predictive Analytics Applications** | Studies employing machine learning (supervised, unsupervised, reinforcement learning), deep learning, natural language processing, predictive modeling, risk stratification algorithms, clinical decision support systems, or data mining techniques to predict outcomes, optimize processes, or support decision-making in healthcare delivery contexts. | • Random forests predicting patient preferences • Gradient boosting for risk stratification • NLP for patient triage • Neural networks for outcome prediction • Ensemble methods for clinical decision support | Comprehensive inclusion of AI/ML techniques recognizes diverse computational approaches with potential to optimize implementation. Focus on predictive applications aligns with review objective while capturing relevant methodological innovations. |

# Table 3. Implementation Science and Outcomes Criteria

| **Criterion** | **Operational Definition** | **Examples of Inclusion** | **Rationale** |
| --- | --- | --- | --- |
| **Implementation Focus (Required)** | Studies must address implementation factors including barriers, facilitators, strategies, adoption processes, sustainability considerations, or system-level integration challenges. Studies focusing solely on clinical efficacy without implementation discussion were excluded. | • Feasibility studies documenting barriers • Pilot studies examining adoption • Implementation trials with process outcomes • Barrier/facilitator analyses • Sustainability assessments | Review focus on implementation optimization requires evidence addressing real-world deployment challenges, not just clinical effectiveness. This criterion ensures included studies contribute actionable implementation insights. |
| **Implementation Outcomes** | Studies examining implementation outcomes as defined by Proctor et al. including acceptability, adoption, appropriateness, feasibility, fidelity, implementation cost, penetration, or sustainability. Clinical outcomes alone insufficient without implementation outcomes. | • Provider adoption rates • Patient acceptability scores • Feasibility metrics (enrollment, completion) • Fidelity to protocol • Cost of implementation | Implementation science framework ensures focus on outcomes relevant to successful real-world deployment beyond clinical effectiveness, critical for translating evidence to practice. |

# Table 4. Study Design and Publication Criteria

| **Criterion** | **Operational Definition** | **Examples of Inclusion** | **Rationale** |
| --- | --- | --- | --- |
| **Eligible Study Designs** | Quantitative (experimental, quasi-experimental, observational), qualitative (interviews, focus groups, ethnography), mixed-methods, systematic reviews, scoping reviews, and substantive theoretical/conceptual papers. Single-arm pilots and feasibility studies included if reporting implementation outcomes. | • Randomized controlled trials • Pilot feasibility studies • Cross-sectional surveys • Qualitative interview studies • Systematic reviews • Implementation frameworks | Scoping review methodology requires broad design inclusion to map full evidence landscape. Inclusion of theoretical papers and reviews enables synthesis of conceptual frameworks alongside empirical findings. |
| **Publication Type** | Peer-reviewed journal articles only. Conference abstracts, dissertations, grey literature, editorials without substantive empirical/theoretical contribution, and protocols excluded. | • Full-text journal articles • Published systematic reviews • Peer-reviewed implementation studies | Peer review ensures methodological quality and validity. Full-text requirement allows adequate extraction of implementation details and contextual factors. |
| **Publication Date** | January 1, 2015 through April 2025 (initial search) and November 2025 (expanded search). This 10-year window captures contemporary AI developments and post-COVID-19 pandemic tele-oncology expansion. | • Studies published 2015-2025 • Recent AI/ML innovations • Post-pandemic telehealth adoption | Starting 2015 captures modern machine learning renaissance and recent telehealth evolution while maintaining contemporary relevance. COVID-19 pandemic fundamentally transformed telehealth landscape, making recent evidence most applicable. |
| **Language** | English-language publications only. Non-English articles excluded due to resource constraints for translation and validation. | • Articles published in English • Studies from any country if published in English | Language restriction is common in scoping reviews. Majority of AI/implementation science literature published in English. Acknowledged as limitation potentially missing relevant international evidence. |

# Table 5. Specific Exclusion Criteria with Examples

| **Exclusion Category** | **Specific Criteria** | **Examples** |
| --- | --- | --- |
| **Clinical Outcomes Focus Without Implementation Considerations** | Studies examining only clinical effectiveness, patient outcomes, or diagnostic accuracy without addressing implementation factors, barriers, or deployment considerations. | • Efficacy trials of telehealth vs. in-person without discussing adoption barriers • AI diagnostic algorithms without implementation context • Survival analyses without delivery model details |
| **Telehealth Outside Oncology Without Clear Transferability** | Non-oncology telehealth studies lacking implementation insights applicable to chronic disease management, complex care coordination, or multi-specialty integration characteristic of cancer care. | • Acute urgent care telemedicine for simple conditions • Mental health teletherapy without relevant barriers • Emergency telemedicine consultation |
| **Insufficient Empirical or Theoretical Contribution** | Opinion pieces, editorials, commentaries without substantive data or theoretical frameworks. Conference abstracts without sufficient methodological detail for assessment. | • Brief conference abstracts • Editorials without data • Perspective pieces without frameworks • Commentaries lacking empirical grounding |
| **Absence of Rural/Implementation Barrier Focus** | Studies that do not address geographic access barriers, implementation challenges, or system-level factors relevant to underserved populations, even if addressing telehealth/AI. | • Telehealth in urban academic centers without barrier discussion • AI models without contextual deployment considerations • Technology development without implementation planning |
| **No AI/Predictive Analytics Application** | Telehealth studies without computational prediction, machine learning, algorithmic decision support, or data-driven optimization components. | • Traditional telehealth without AI enhancement • Descriptive studies without predictive modeling • Simple dashboards without ML algorithms |
| **Inadequate Methodological Detail** | Studies lacking sufficient description of methods, setting, population, intervention, or outcomes to assess relevance or extract meaningful implementation insights. | • Vague descriptions of AI methods • Unclear setting or population characteristics • Missing barrier/facilitator details • Insufficient outcome reporting |

# Screening Process and Decision Rules

## Two-Stage Screening

1. Title/Abstract Screening: Two independent reviewers (RZ, MM) assessed each record against inclusion criteria. Liberal threshold applied: studies advanced if ANY inclusion criterion potentially met. Cohen's κ = 0.78 (substantial agreement). 2. Full-Text Review: Same reviewers independently assessed full articles against complete criteria. Detailed documentation of exclusion reasons required. PI (LH) resolved all conflicts through consensus discussion. Cohen's κ = 0.82 (substantial agreement).

## Hierarchy of Exclusion Reasons

When multiple exclusion criteria applied, primary reason recorded as: 1. Publication type (conference abstract, protocol) 2. No implementation focus (clinical outcomes only) 3. Non-oncology without transferability 4. No AI/predictive analytics 5. Insufficient methodological detail 6. Language/date restrictions

## Edge Cases and Resolution

| **Ambiguous Case** | **Resolution Approach** |
| --- | --- |
| Non-oncology study with potentially relevant implementation barriers | Included if barriers clearly applicable to chronic disease management, multi-specialty coordination, or complex care characteristic of oncology |
| Study with minimal AI application | Included if employing any computational prediction or algorithmic decision support, even if not primary focus |
| Systematic review including some relevant studies | Included if synthesis provided implementation insights beyond individual study summaries. Citation chased for primary studies |
| Urban setting study without explicit rural focus | Included if addressing access barriers, underserved populations, or implementation challenges transferable to rural contexts |

# Quality Assessment

Formal quality assessment was not conducted, consistent with established scoping review methodology. Scoping reviews prioritize evidence mapping and gap identification over intervention effectiveness synthesis, which is the purview of systematic reviews requiring quality appraisal. However, study design characteristics and methodological limitations were extracted and are noted in Table 1 of the main manuscript to contextualize evidence quality.

# Documentation and Transparency

All screening decisions were documented in Covidence systematic review software with:

• Individual reviewer decisions (include/exclude/maybe) • Specific exclusion reasons for each rejected article • Conflict resolution notes for discrepant decisions • Full-text page numbers where criteria were assessed • Final disposition for all 330 screened records. This documentation enables full reproducibility and transparency of selection processes, consistent with PRISMA-ScR reporting standards.
