# Supplementary material for: AI-Enhanced Predictive Analytics to Optimize Tele-Oncology Implementation in Rural Settings: Scoping Review
Source: JMIR Cancer. 2026 Jul 16;12:e78005. doi: 10.2196/78005 (PMC13374798; doi:10.2196/78005)
Supplement: Multimedia Appendix 3 [file cancer-v12-e78005-s003.docx]

**Supplementary File 4: Data Extraction Form**

# Instructions

Reviewer ID: _________ Study ID: _________ Date: _________

*Complete all applicable fields. If information is not reported, write "NR" (not reported). For unclear information, write "unclear" and note details in Comments section. Extract data verbatim when possible; paraphrase minimally when necessary for clarity.*

# Section 1: Study Characteristics

| **Field** | **Extracted Data** |
| --- | --- |
| Author(s) |  |
| Year of Publication |  |
| Country/Countries |  |
| Study Design (e.g., RCT, pilot study, cross-sectional, qualitative, systematic review) |  |
| Setting/Healthcare System (e.g., academic medical center, community hospital, VA, multi-site) |  |
| Geographic Setting (rural, urban, mixed; specify rurality definition if provided) |  |
| Study Duration/Timeframe |  |

# Section 2: Population Characteristics

| **Field** | **Extracted Data** |
| --- | --- |
| Sample Size (total N) |  |
| Population Type (patients, providers, both, other stakeholders) |  |
| Cancer Type/Oncology Focus (if applicable; specify cancer type or "general oncology") |  |
| Key Demographics (age, sex, race/ethnicity if reported) |  |

# Section 3: Telehealth/Tele-oncology Implementation

| **Field** | **Extracted Data** |
| --- | --- |
| Telehealth Modality (video, telephone, remote monitoring, store-and-forward, hybrid) |  |
| Clinical Application (consultation, monitoring, diagnosis, treatment planning, follow-up) |  |
| Technology Platform/System (describe platform, vendor if mentioned) |  |

# Section 4: AI/Predictive Analytics Applications

| **Field** | **Extracted Data** |
| --- | --- |
| AI/ML Technique(s) (e.g., random forest, gradient boosting, neural network, NLP, logistic regression) |  |
| Prediction Target/Outcome (what does the model predict?) |  |
| Input Features/Variables (what data feeds the model?) |  |
| Model Performance Metrics (accuracy, AUC, sensitivity, specificity, etc.) |  |
| Validation Approach (cross-validation, external validation, none) |  |

# Section 5: Implementation Factors

| **Field** | **Extracted Data (list all identified)** |
| --- | --- |
| Implementation Barriers (specify level: patient, provider, organizational, system) | Patient-level:  Provider-level:  Organizational-level:  System-level: |
| Implementation Facilitators (specify level: patient, provider, organizational, system) | Patient-level:  Provider-level:  Organizational-level:  System-level: |
| Implementation Strategies Used (if described) |  |
| Implementation Framework Used (e.g., CFIR, RE-AIM, EPIS, or none specified) |  |

# Section 6: Outcomes

| **Field** | **Extracted Data** |
| --- | --- |
| Implementation Outcomes (acceptability, adoption, appropriateness, feasibility, fidelity, penetration, sustainability, cost) |  |
| Clinical Outcomes (if reported) |  |
| Process Measures (utilization rates, adherence, engagement) |  |

# Section 7: Key Limitations and Notes

| **Field** | **Extracted Data** |
| --- | --- |
| Key Methodological Limitations (as noted by authors or apparent to reviewer) |  |
| Funding Source (as reported) |  |
| Additional Notes/Comments (reviewer observations, uncertainties, items for PI review) |  |

*Form completed by: _______________________ Date: _____________*

*Reviewed/verified by PI: _______________________ Date: _____________*
