# Supplementary material for: AI-Enhanced Predictive Analytics to Optimize Tele-Oncology Implementation in Rural Settings: Scoping Review
Source: JMIR Cancer. 2026 Jul 16;12:e78005. doi: 10.2196/78005 (PMC13374798; doi:10.2196/78005)
Supplement: Checklist 2 [file cancer-v12-e78005-s005.docx]

**Supplementary File 2**

**PRISMA for Literature Searches (PRISMA-S) Checklist**

*Manuscript: AI-Enhanced Predictive Analytics to Optimize Tele-Oncology Implementation in Rural Settings: A Scoping Review*

Reference: Rethlefsen ML, Kirtley S, Waffenschmidt S, et al. PRISMA-S: an extension to the PRISMA statement for reporting literature searches in systematic reviews. Syst Rev. 2021;10(1):39. doi: 10.1186/s13643-020-01542-z

| **Section/Topic** | **#** | **Checklist item** | **Reported on page #** | **Notes** |
| --- | --- | --- | --- | --- |
| **REPORTING THE SEARCH — DATABASE SEARCHING** | | | | |
| Database name | 1 | Name each individual database searched. | Page 5 |  |
| Platform/interface name | 2 | Name the platform or interface used, if applicable. | Page 5 |  |
| Coverage dates | 3 | State the date coverage of each database searched. | Page 5 |  |
| **REPORTING THE SEARCH — SEARCH STRATEGIES** | | | | |
| Full search strategies | 4 | Provide the full search strategy for all databases, interfaces, and platforms searched, including all search terms used, Boolean operators, truncation, wildcards, field tags, limits, and date restrictions. | Supplementary File 2 |  |
| **REPORTING THE SEARCH — SEARCH METHODS** | | | | |
| Search filters | 5 | Identify and describe any validated or established search filters used, providing citations and/or full strategies. | Page 5 |  |
| Limits and restrictions | 6 | Describe any limits applied, such as date limits, language limits, and publication type limits. | Page 5 |  |
| Search updates | 7 | Indicate whether searches were updated from the initial date to the final inclusion decision date, and if so, provide the updated date of search. | Page 5 |  |
| **REPORTING THE SEARCH — OTHER SEARCH METHODS** | | | | |
| Effort to retrieve grey literature | 8 | Describe the effort to search for grey literature, including unpublished studies, conference abstracts, and dissertations. | Page 5 |  |
| Databases and sources for other types of evidence | 9 | Name any databases or sources searched for other types of evidence (e.g., trial registers, guidelines databases). | N/A |  |
| Cited reference searching | 10 | Describe any efforts to search for cited or citing references. | Page 5 |  |
| Website and organization searching | 11 | Describe any searches of websites and organizations. | N/A |  |
| Contacting experts | 12 | Describe any efforts to contact experts. | N/A |  |
| **REPORTING THE SEARCH — INFORMATION ABOUT SEARCHES** | | | | |
| Dates of searches | 13 | For each source, provide the most recent date searched. | Page 5 |  |
| Peer review of the search strategy | 14 | Indicate whether the search strategies were peer reviewed, and if so, describe the process. | Page 5 |  |
| Deduplication | 15 | Name the software or process used to manage records identified in the search, and describe the deduplication process. | Page 6 |  |
| Total records identified | 16 | Provide the total number of records identified from each database and through other methods. | Page 7, Figure 1 |  |
